# An Umbrella Review of the Best and Most Up-to-Date Evidence on the Built Environment and Physical Activity in Older Adults ≥60 Years

**DOI:** 10.3389/phrs.2023.1605474

**Published:** 2023-03-10

**Authors:** Jodie A. Stearns, Hayford M Avedzi, Desmond Yim, John C. Spence, Farshad Labbaf, Carminda G. Lamboglia, Fiona Ko, Ciara Farmer, Ellina Lytvyak, Megan Kennedy, Yeong-Bae Kim, Hui Ren, Karen K. Lee

**Affiliations:** ^1^ Division of Preventive Medicine, Department of Medicine, Faculty of Medicine and Dentistry, University of Alberta, Edmonton, AB, Canada; ^2^ Faculty of Kinesiology, Sport, and Recreation, University of Alberta, Edmonton, AB, Canada; ^3^ University of Alberta Library, University of Alberta, Edmonton, AB, Canada

**Keywords:** built environment, older adults, umbrella review, physical activity, walking, biking

## Abstract

**Objectives:** To present the best and most up-to-date evidence on associations between built environment (BE) attributes and overall and specific domains of physical activity (PA) (i.e., leisure, transport, walking, and cycling) in older adults (≥60 years).

**Methods:** An umbrella review was undertaken to compile evidence from systematic reviews using the Joanna Briggs Institute methodology. A comprehensive search (updated 16 August 2022), inclusion/exclusion of articles *via* title/abstract and full-text reviews, data extraction, and critical appraisal were completed. Only reviews with a good critical appraisal score were included.

**Results:** Across three included systematic reviews, each BE attribute category was positively associated with ≥1 PA outcome. A larger number of significant associations with BE attributes were reported for transport walking (13/26), total walking (10/25), and total PA (9/26), compared to leisure walking (4/34) and transport cycling (3/12). Fewer associations have been examined for leisure cycling (1/2).

**Conclusion:** Although the causality of findings cannot be concluded due to most primary studies being cross-sectional, these best and most up-to-date findings can guide necessary future longitudinal and experimental studies for the (re)design of age-friendly communities.

## Introduction

Regular physical activity (PA), defined as physical movement involving energy expenditure during work, play, chores, travel, and recreation [1, 2], is fundamental to healthy aging. Physically active older adults, defined here as people 60 years or older [3, 4], retain independence and functional capacity much longer [5, 6], enjoy better prevention and management of chronic diseases like diabetes and cardiovascular diseases [7, 8], experience less stress, have lower rates of depression, anxiety, and social isolation [9, 10], and have boosted immune responses when faced with illness compared to those who are inactive [11, 12]. Conversely, those who reduce their PA during their older years are more likely to experience rapid health declines, loss of functional independence, and poor quality of life, leading to increased healthcare costs [13, 14]. Unfortunately, many older adults globally do not engage in sufficient levels of PA; for example, 17% of Canadian adults aged 60 to 79 meet the minimum recommendation of 150 min of moderate-to-vigorous PA per week [15]. To promote healthy aging, it is crucial to understand how best to support older adults to be physically active.

The built environment (BE), defined as human-created physical aspects of the environment (e.g., buildings, parks, neighbourhoods) [16], is thought to play an important role in heathy aging [17]. Macro-scale BE features include neighbourhood-level structural features (e.g., street connectivity, density, mixed land use) whereas micro-scale BE features include smaller street-level features (e.g., benches, light, sidewalk conditions), and both have differential effects on physical activity [18, 19]. Older adults may experience unique challenges to interacting with their physical environment, including physical and psychological barriers such as frailty, fear of falls, and concerns around safety from long distances to destinations, steep sidewalk slopes, high traffic-speed, and crime [20, 21]. The BE may also be particularly important for older adults who lose the ability to drive and may become more reliant on their immediate neighbourhood environment, travel shorter distances from home, and rely on active transportation modes [22]. Additionally, older adults may prefer indoor PA such as climbing indoor stairs and walking in hallways (e.g., of retirement community apartments) and malls as safe modes of exercise [23, 24]. Thus, BE building features may also support or hinder PA for those living in these communities [25]. Identifying factors within different levels of the BE—including buildings, outdoor spaces, and neighbourhoods—that promote PA in older adults is important for informing design of age-friendly interventions for both testing and implementation in real world settings [26].

Umbrella reviews provide an overall assessment of the literature on a topic by integrating and summarizing findings from systematic reviews [27]. According to a recent umbrella review by Prince et al. [28], total PA in older adults (≥65 years) is associated with forest/trees, recreation facilities, walkability, pedestrian safety infrastructure, comfort infrastructure (e.g., benches), urbanization, and senior residence design; leisure PA is associated with parks/playgrounds; and transport PA is associated with walkability, cycling infrastructure, urbanization, and neighbourhood characteristics. Another umbrella review by Bonaccorsi et al. [29] identified several correlates of overall PA in older adults (≥55 years), with more BE correlates for transport compared to leisure PA. However, these umbrella reviews did not provide an overview of the best and most up-to-date evidence by: 1) ensuring the included reviews met the criteria of a systematic review, 2) using study quality as a secondary exclusion criterion, and 3) restricting the publication of reviews to the past 10 years.

This umbrella review provides a concise overview of the best and most up-to-date evidence from good quality systematic reviews on BE attributes associated with overall and specific domains of PA (i.e., leisure, transport, walking, cycling) in older adults (≥60 years). Only reviews that met the criteria of a systematic review [30], received a good quality assessment score, and were published in the last 10 years were included.

## Methods

The review was guided by the Joanna Briggs Institute (JBI) methodology for conducting umbrella reviews [31], and other umbrella review [32, 33] and systematic review recommendations [34]. It follows the *Preferred Reporting Items for Systematic Reviews and Meta-Analyses (PRISMA)* [35], along with a study protocol.

### Data Sources and Search Strategy

The literature search was developed by an experienced health sciences librarian who is a member of the research team. Database searches were performed in July 2020 in: MEDLINE *via* OVID (1946 - 30 July 2020), EMBASE *via* OVID (1974 - 30 July 2020), Cumulative Index to Nursing and Allied Health Literature (CINAHL) Plus with Full Text *via* EBSCOhost (1936 - 30 July 2020), Scopus *via* Elsevier (1970 - 30 July 2020), The Cochrane Library *via* Wiley (1992 - 30 July 2020), and Environment Complete *via* EBSCOhost (1897 - 30 July 2020). Searches used a combination of natural language vocabulary and controlled terms (subject headings) [36]. Natural language terms were derived from three main concepts: 1) built environment, such as city planning, urbanization, transportation, architecture, and design; 2) PA, such as walking, cycling, exercise, and leisure PA; and 3) older adults (Supplementary File S1). The search was limited to reviews on quantitative or qualitative studies including systematic, scoping, integrative, rapid, umbrella, meta-analyses, health technology assessments, and other types of reviews. To increase sensitivity of the search, language, publication date, or other limits were not applied. The search was updated in February 2021 and August 2022.

Reference lists of included reviews and umbrella reviews on similar topics were searched. Using Google Scholar, a basic keyword search was conducted to identify reviews that may have been missed, along with articles citing included reviews (4 pages of results were searched). Finally, major repositories of systematic reviews, including JBI Database of Systematic Reviews and Implementation Reports, the Cochrane Database of Systematic Reviews, DARE, and the PROSPERO register were searched.

### Criteria for Study Consideration

Reviews were included if they synthesized/summarized findings on the association between BE attributes and PA in older adults, met the PECOS criteria [33] described in the following, and were published after 2011 (reflecting the past 10 years as recommended by JBI methodology), available in full-text, and written in English.

#### Population

Older adults are defined as individuals ≥60 years [3, 4]. Systematic reviews that included other age groups (e.g., adults <60 years old) were included if findings stratified and synthesized/summarized results for older adults ≥60 years old. This umbrella review focused on reviews of community-dwelling older adults and excluded reviews exclusively focused on clinical populations (e.g., overweight or diabetic populations), and reviews with studies that included participants needing advanced or long-term care, or with major neurocognitive disorders that interfere with PA.

#### Exposure

The exposure of interest was BE attributes or change in BE attributes. The BE was defined as a human-made physical environment such as buildings, parks, land use patterns, transportation infrastructure, and community layout and design [16]. Multiple-component reviews were included if BE factors and their association with PA outcomes among older adults were core components [37].

#### Comparator(s)

Included reviews could compare exposures (BE attributes) of interest to alternative exposures (e.g., no exposure to BE intervention, lower level of exposure to BE intervention, standard care/practice).

#### Outcome

The outcome of interest was PA or change in PA (e.g., frequency, duration, intensity, type), defined as physical movement requiring energy expenditure—including activities undertaken while working, playing, carrying out household chores, travelling, and engaging in recreational pursuits [1, 2, 38]. Specific outcomes of interest included overall and specific domains of physical activity (i.e., leisure, transport, walking, and cycling).

#### Study Design

Peer-reviewed, published systematic reviews (with or without meta-analysis) of quantitative primary studies were eligible for inclusion. They had to include the following defining features of a systematic review to be included: i) a research question; ii) a reproducible and complete search strategy (i.e., description of databases/platforms/engines searched, search dates) iii) inclusion and exclusion criteria; iv) methods used to screen and select articles; v) a critical appraisal and summary of study quality (e.g., risk of bias); and vi) a reproducible summary of the data analysis and synthesis of results [30]. Theoretical and opinion-based reviews, scoping reviews, reviews of qualitative research, literature reviews, and umbrella reviews were excluded. Grey literature reviews were not included as they are not peer-reviewed and thus we cannot be confident that they followed rigorous scientific procedures [39, 40]. No limitations were placed on the design of primary studies included in the reviews. Reviews with critical appraisal scores of at least 6 out of 11 were included.

### Screening and Critical Appraisal

Two authors independently screened titles and abstracts within the Covidence software and conflicts were resolved by consensus. Two authors independently screened full‐text reviews for inclusion criteria; conflicts were resolved by consensus. Unresolved conflicts were resolved with the involvement of a third author. Following the full‐text reviews, reasons for exclusion were recorded. Two authors independently critically appraised the included reviews using the JBI Critical Appraisal Checklist [41]. The screening and critical appraisal process was completed by several team members all with research experience and graduate degrees.

### Data Extraction

Following study protocol, data extraction was conducted independently by at least two reviewers using a structured form in Microsoft Excel (Microsoft Corporation 2018). Based on the JBI methodology, the following information was extracted: citation details, objective, if a meta-analysis was performed, participant characteristics, setting and context, number of searched databases, date range of searches, publication date ranges, continents/countries, types of study designs, instruments used for quality appraisal, BE attributes assessed, PA outcomes, BE and PA measurement details, and method of synthesis/analysis. Findings showing associations between the BE attributes and overall and specific domains of PA (i.e., leisure, transport, walking, and cycling) were extracted.

### Data Summarization

Relevant findings were organized and summarized by BE attribute and PA outcome. No additional synthesis of the data was performed.

BE attribute categories were based on the Neighbourhood Environment Walkability Scale (NEWS) [42, 43] and included walkability, residential density and urbanization, street connectivity, access to and/or availability of services and destinations, pedestrian and/or cycling infrastructure and streetscape, aesthetics and cleanliness and order, and safety and traffic. Specific BE attributes were organized within these features.

As recommended by the JBI methodology, overlap in primary studies was addressed [41]. It was calculated using the corrected covered area (CCA) [44], for which >15% indicates very high overlap, 10%–15% indicates high overlap, 5%–10% indicates moderate overlap, and <5% indicates low overlap [32]. Based on the overlap score, decisions were made on how to deal with the overlap (e.g., create citation matrices by PA outcome, exclude reviews with a high degree of overlap).

## Results

**FIGURE 1:**
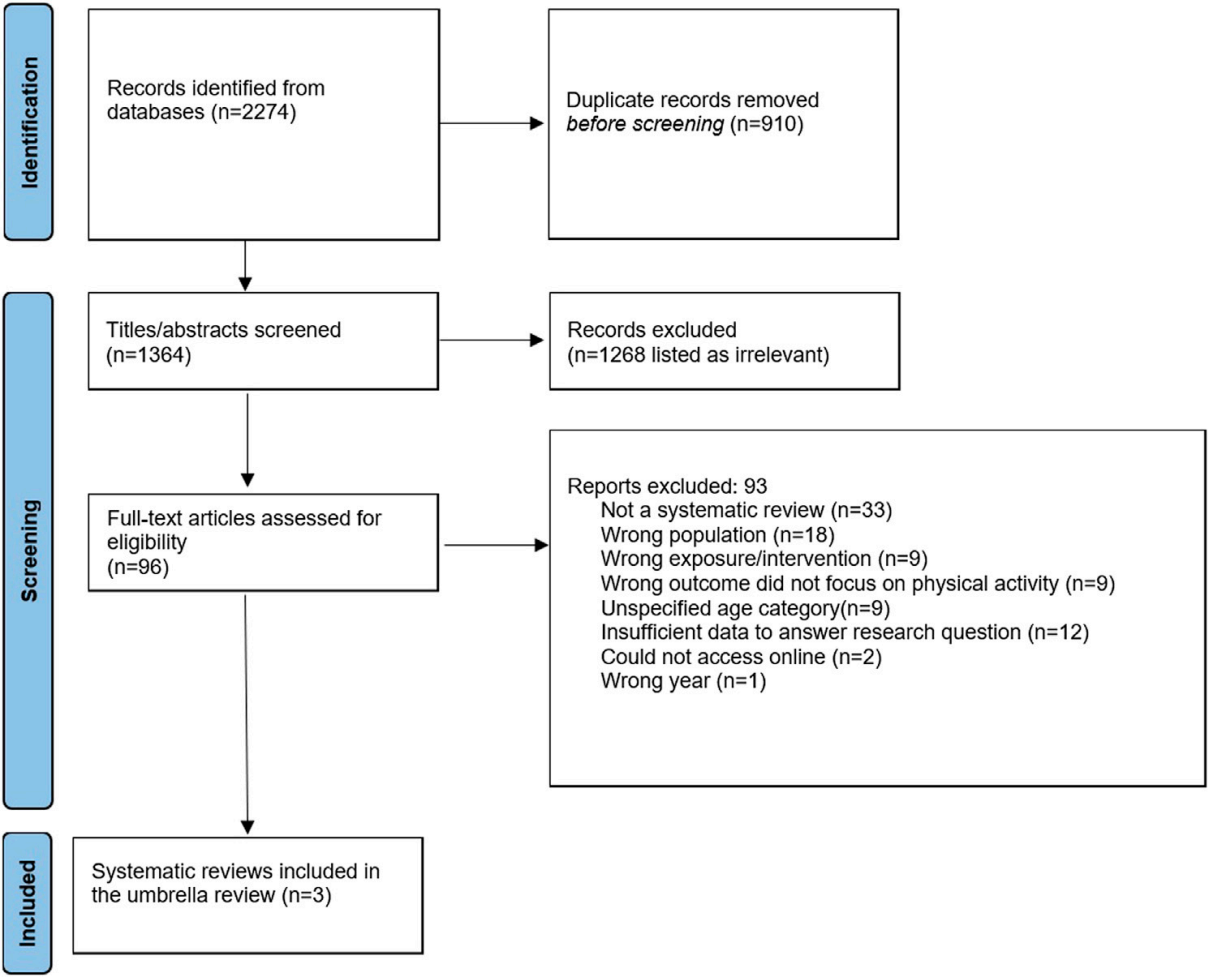
Preferred Reporting Items for Systematic Reviews and Meta-Analyses flow diagram (Global, 2001–2016).

A PRISMA flow diagram of the systematic literature search and results is presented in Figure 1. There were 2274 references identified, and 910 duplicates removed. After screening 1364 titles and abstracts against the inclusion criteria, a further 1268 were excluded. There were 96 reviews assessed for full‐text eligibility, and a further 93 documents were excluded for reasons including unspecified age categories, non-systematic reviews, incorrect population, and non-BE interventions and exposures. Several systematic reviews in particular were excluded because they did not report a summary/synthesis of findings across studies for older adults [45–47], did not report a summary of the quantitative findings specifically [23], or did not meet the criteria of a systematic review [48, 49]. Ultimately, three reviews met the inclusion criteria [42, 50, 51].

### Characteristics of Included Reviews

The three included systematic reviews all had meta-analyses and were published between 2017–2018, and included 42–100 primary studies published between 2000 and 2017 (see Table 1) [42, 50, 51]. Most primary studies included in the reviews were cross-sectional (between 94%–100%), and a few were longitudinal (between 0%–5%). One quasi-experimental study was identified in one review [50]. Reviews included primary studies from countries on all continents, however most studies were from North America and European countries.

**TABLE 1:** Characteristics of included systematic reviews (Global, 2001–2016).

Reference	Meta-analysis	Critical appraisal score	Objective	Number of primary studies	Search period	Primary study publication dates	Continents/Countries	Study design	Built environment attributes	Physical activity outcomes
[50]	Yes	11	To provide a timely, robust overview of studies that investigated associations of BE attributes and estimates of total PA, including total walking	100	2000–2016	2001–2016	Australia, Belgium, Brazil, Canada, China, Colombia, Czech Republic, Hong Kong, Iceland, Iran, Japan, Lithuania, Malaysia, Netherlands, Norway, Poland, Slovakia, Singapore, South Africa, South Korea, Thailand, UK, USA	• Cross-sectional (94%)	6 BE attribute categories:	• Total PA^a^
								• Longitudinal (5%)	• Walkability	• Total walking^a^
								• Quasi-experimental (1%)	• Residential density/urbanisation	Studies measured PA using self-report and/or objective measures
									• Street connectivity	
									• Access to/availability of destination and services	
									• Infrastructure and streetscape	
									• Safety	
									26 total BE attributes	
									Studies measured BE attributes using perceived (48%) or objective measures (37%), or both (13%)	
[42]	Yes	11	To systematically review the literature on neighbourhood physical environmental correlates of active travel in older adults	42	2000–2016	2004–2016	Africa, Asia, Europe, North America, Oceania, South America	• Cross-sectional (100%)
									7 BE attribute categories:	• All active travel
									• Residential density/urbanisation	• Total walking for transport^a^
									• Walkability	• Within-neighbourhood walking for transport
									• Street connectivity	• Combined walking and cycling for transport
									• Access to/availability of services/destinations	• Cycling for transport^a^
									• Pedestrian and cycling infrastructure	All studies measured PA using self-reported instruments
									• Aesthetics and cleanliness/order	
									• Safety and traffic 26 total BE attributes Studies measured BE attributes using perceived (43%) or objective measures (38%), or both (14%)
[51]	Yes	11	To systematically review and quantitatively summarise studies on relationships between physical environmental attributes and leisure-time PA among older adults, including those from grey literature Sources	72	2000–2017	2006–2016	North America, East Asia and Pacific, Europe, Central Asia, Latin America and Caribbean, Sub-Saharan Africa, Middle East and North Africa, South Asia	• Cross-sectional (99%)	7 BE attribute categories:	• Leisure-time walking^a^
								• Longitudinal (1%)	• Walkability	• Leisure-time walking within the neighbourhood
									• Residential density/urbanisation	• Leisure-time cycling^a^
									• Street connectivity	• Leisure-time walking and cycling combined
									• Access to/availability of services/destinations	• Overall leisure-time PA
									• Pedestrian/cycling infrastructure and streetscape	• Leisure-time PA other than walking
									• Aesthetics and cleanliness/order	All studies measured PA using self-reported instruments
									• Safety and traffic 34 total BE attributes Studies measured BE attributes using perceived (42%) or objective measures (44%), or both (14%)	

^a^ Indicates variables included in our umbrella review.

BE, built environment; PA, physical activity.

Reviews reported between 6 and 7 BE attribute categories and presented findings for 26 to 34 total BE attributes. All reviews included primary studies using objective (37%–44%; e.g., geographical information systems) and/or perceived (42%–48%; e.g., questionnaires) BE measurements or both (13%–14%).

Reviews summarized results for up to 6 PA outcomes along with differences by BE measurement (perceived vs. objective) [42, 50], PA measurement (self-reported vs. objective) [50], and effect moderators [42, 50, 51]. However, only results directly pertaining to the research objective of this umbrella review are included. Specifically, the results for total PA (31 studies) and total walking (55 studies) were included from Barnett et al. [50], results for leisure walking (34 studies) and leisure cycling (2 studies) were included from Van Cauwenberg et al. [51], and results for transport walking (35 studies) and transport cycling (2 studies) were included from Cerin et al. [42]. Leisure and transport outcomes were measured *via* self-report [42, 51], and total PA and total walking were measured *via* objective (e.g., accelerometers) and self-report (e.g., questionnaires) [50].

The CCA indicated 16% overlap in the primary studies from the three reviews, which is considered very high [32, 44]. However, because each review focused on distinct PA outcomes, the overlap in specific findings was zero as there is only one review per outcome. No further steps were completed to deal with the overlap.

### Quality of Included Reviews

Using the JBI critical appraisal tool [41], methodological quality for all reviews was 11/11 (Supplementary File S2) [42, 50, 51].

### Summary of Main Findings

The summary of meta-analysis findings for associations between different BE attributes and the 6 PA outcomes for older adults is presented in Table 2. In each of the three reviews [42, 50, 51], a “traditional” meta-analysis was not possible due to the variety of different BE and PA measures used in the primary studies. Instead, the authors quantitively synthesized findings by using a conservative meta-analytic approach, giving greater weight to studies of higher quality and larger sample sizes [50]. Using this procedure, the authors identified significant positive associations (P), significant negative associations (N), and non-significant findings (Ø). Unless otherwise indicated, significant findings (*p* ≤ 0.05) presented within the text are considered sufficiently studied in ≥5 primary studies. There are 26 findings for total PA, 25 findings for total walking, 26 findings for transport walking, 12 findings for transport cycling, 34 findings for leisure walking, and 2 findings for leisure cycling.

**TABLE 2:** Umbrella review findings of associations between built environment attributes and physical activity in older adults (≥60 years; Global, 2001–2016).

Built environment categories and attributes	Total physical activity (50)	Total walking (50)	Transport walking (42)	Transport cycling (42)	Leisure walking (51)	Leisure cycling (51)
Walkability	**P**	**P**	**P**	Ø	**P**	Ø
Residential density and urbanisation	Ø	**P**	**P**	N	Ø	
Street connectivity	Ø	Ø	**P**		Ø	
Access to/availability of services/destinations						
Overall access to destinations and services	**P**	**P**	**P**	Ø		
Land-use mix—access					**P**	
Land-use mix—destination diversity	Ø	Ø	**P**		Ø	
Shops/commercial/services	**P**	**P**	**P**	P	Ø	
Food outlets	Ø	Ø	Ø			
Business/government/institutional/industrial			Ø			
Government/financial services	Ø	Ø				
Institutional/industrial					Ø	
Education facilities	Ø	Ø				
Health and aged-care	Ø	Ø	Ø		Ø	
Religious	Ø	Ø	Ø			
Community centre					Ø	
Entertainment			Ø		Ø	
Public transit	**P**	**P**	**P**	P	Ø	
Recreational facilities	**P**	Ø			Ø	
Walking/cycling facilities						
Social recreational facilities	Ø	Ø				
Gym/fitness facilities					Ø	
Swimming pool					Ø	
Park/open space	**P**	**P**	**P**		Ø	
Playground					Ø	
Outdoor sports field					Ø	
Other destinations	Ø		Ø			
Pedestrian/cycling infrastructure and streetscape						
Access to cycle/walk-friendly infrastructure	Ø	Ø				
Walk-friendly infrastructure	**P**	**P**	**P**			
Cycle-friendly infrastructure	Ø	Ø				
Footpaths/Pavement presence/quality	Ø	Ø			Ø	
Other infrastructure for walking/cycling					Ø	
Slopes/hilliness					Ø	
Barriers to walking/cycling	Ø	Ø	Ø		Ø	
Easy access to building entrance			P		Ø	
Indoor places for walking					Ø	
Benches/sitting facilities			**P**	Ø	Ø	
Streetlights	Ø	P	Ø	Ø	Ø	
Public toilets			Ø	Ø	Ø	
Aesthetics and cleanliness/order						
Greenery and aesthetically pleasing scenery	**P**	**P**	Ø	Ø		
Greenery					Ø	
Aesthetically pleasing scenery					**P**	
Littering/vandalism/decay/vacant buildings			**N**	Ø	Ø	
Pollution (air, noise, sewer)	Ø	Ø	Ø	Ø	Ø	
Safety and traffic						
Crime/personal safety	**P**	**P**	Ø		Ø	P
Traffic/pedestrian safety	Ø	Ø	Ø	Ø	Ø	
Human or motorised traffic volume			P		Ø	
General safety					P	

P, positive significant association based on the results of meta-analyses (*p* ≤ 0.05); N, negative significant association based on the results of meta-analyses (*p* ≤ 0.05); Ø, nil association/non-significant (*p* > 0.05), bolded significant findings indicate the association was sufficiently studied in ≥5 studies.

#### Walkability

Walkability was positively associated with 4 of the PA outcomes, including total PA, total walking, transport walking, and leisure walking.

#### Residential Density and Urbanization

Residential density and urbanization were positively associated with total walking, and transport walking. Transport cycling was negatively associated with residential density and urbanization but was insufficiently studied (<5 studies).

#### Street Connectivity

Street connectivity was positively associated with transport walking.

#### Access to/Availability of Services/Destinations

Overall access to destinations and services was positively associated with total PA, total walking, and transport walking. Land-use mix (access) was positively associated with leisure walking, and land-use mix (destination diversity) was positively associated with transport walking.

Regarding access to/availability of specific types of destinations, at least one of the studied destinations was associated with total PA, total walking, transport walking, and transport cycling. Access to/availability of shops/commercial/services and public transit were positively associated with total PA, total walking, transport walking, and although not sufficiently studied (<5 studies), were positively associated with transport cycling. Access to/availability of recreational facilities were positively associated with total PA, and access to/availability of parks/open spaces were positively associated with total PA, total walking, and transport walking.

#### Pedestrian/Cycling Infrastructure and Streetscape

Pedestrian infrastructure and streetscape BE attributes were positively associated with total PA and walking, and transport walking. Specifically, walk-friendly infrastructure was positively associated with total PA, total walking, and transport walking. Benches/sitting facilities were positively associated with transport walking. Although not sufficiently studied (<5 studies), easy access to building entrances was positively associated with transport walking. Streetlights were positively associated with total walking.

#### Aesthetics and Cleanliness/Order

BE correlates of aesthetics and cleanliness/order were identified for total PA, total walking, transport walking, and leisure walking. Greenery and aesthetically-pleasing scenery were positively associated with total PA and total walking, and aesthetically-pleasing scenery was positively associated with leisure-time walking. Littering/vandalism/decay/vacant buildings were negatively associated with transport walking.

#### Safety and Traffic

There were five PA outcomes associated with safety and traffic BE attributes. Specifically, crime and personal safety was positively associated with total PA and total walking. Although insufficiently studied (<5 studies), human or motorized traffic volume, general safety, and crime/personal safety were associated with transport walking, leisure walking, and leisure cycling, respectively.

#### Additional Findings

Although not a part of the findings for this umbrella review, the reviews did find some associations to be dependent on the type of PA measure (i.e., objective vs. self-report [50]), and type of BE measure (i.e., objective vs. perceived [42, 50]). Finally, several individual and environmental moderators were also examined in the three reviews, but the results were inconsistent.

## Discussion

This umbrella review summarizes the best and most up-to-date evidence on associations between BE attributes and PA in older adults (≥60 years). Three systematic reviews (with meta-analyses) of good methodologic quality were included. BE attributes were associated with each of the PA outcomes examined including total PA (9/26 significant associations), total walking (10/25 significant associations), transport walking (13/26 significant associations), transport cycling (3/12 significant associations), leisure walking (4/34 significant associations), and leisure cycling (1/2 significant associations). Each of the BE categories, including walkability, residential density and urbanization, street connectivity, access to/availability of services/destinations, pedestrian/cycling infrastructure and streetscape, aesthetics and cleanliness/order, and safety and traffic were associated with at least one of the PA outcomes. There was no overlap of primary studies by outcome.

The observed importance of walkability, a macro-scale BE feature that includes street connectivity, land-use mix, and density, for total PA and walking (total, transport, leisure) is consistent with a scoping review of reviews, which identified walkability as the BE factor most consistently associated with PA in all populations [52]. Thus, having walkable streets appears to be a universally important feature across the lifespan.

Having access to/availability of services/destinations was also observed to be an important feature for total PA, total walking, and transport walking. This is consistent with a review of qualitative studies that highlighted access to general shops and services, such as grocery stores, libraries, mailboxes, newspaper-boxes, post offices, senior-oriented amenities, and public transit playing a role for older adults in choosing to walk [21]. Since shopping is a major reason for older adults to leave home [53], having access to important destinations close to home appears to encourage older adults to actively commute, contributing to total levels of PA.

Presence of walk-friendly infrastructure was found to be important for total PA, total walking, and transport walking. Benches or sitting facilities was associated with transport walking. Findings from a qualitative review [21], showed that older adults preferred streets with: sidewalks; strategically-placed curb cuts and handrails; available benches and clean public washrooms; and street lighting. They disliked: abrupt endings of sidewalks; steep gradients or stairs; cracked, uneven, steep, or high curbs; ice and snow; inadequate separation between pedestrians and other forms of active transport; zebra crossings with unclear indicators of pedestrian crossings; long crossing distances across multiple lanes; and inadequate signal times.

Presence of greenery and aesthetically-pleasing scenery was important for total PA, total walking, and leisure walking. According to Moran et al. [21], the aesthetic appeal of both private and public properties plays important roles in PA and walking. Aesthetic appeal of spaces transforms the objective environment into a subjective experience for community members [54], and hence may promote PA and walking. This was highlighted in interviews by Zandieh et al. [55], where older adults stated cleanliness, presence of greenery, and natural landscapes provided them with the incentive to walk outdoors. In addition to green spaces, aesthetic appeal can involve the design of white spaces (environmental snow and ice [54]), through the use of site planning, landscaping, and evergreen vegetation [56].

Crime and personal safety were identified as important for total PA and total walking. The qualitative review by Moran et al. [21] also identified perceptions of safety as playing a role in walking. Safety derived from the BE that can impact PA of older adults includes safety from crime, traffic, and falling [21, 55]. Safety from crime can be derived from proxies of safety (e.g., graffiti, litter, vandalism, abandoned buildings, and street lighting) [57] and the presence of different groups of people (e.g., presence of perceived-to-be socially-responsible persons versus other perceived-to-be threatening groups like intimidating groups of youths) [21]. Presence and quality of pedestrian facilities, such as auditory and visual signals for street crossing, quality of sidewalks, walking trails, crosswalks, and benches, provide safety from fall injury and traffic [58, 59]. Street calming features (e.g., curb extensions, medians, raised speed reducers, inclusion of bike lanes) can also prevent pedestrian road traffic injury and promote pedestrian activity [60, 61].

Of particular note is that building BE features were understudied, with “easy access to building entrance” (positively associated with transport walking but insufficiently studied) and “indoor places for walking” only having findings for transport and leisure walking. This highlights the lost opportunity of also using indoor environments in the promotion of PA for older adults. As many older adults have a fear of falls from being active outdoors, especially in winter climates, having safe and accessible spaces to be active indoors such as in the hallways of apartments and malls are particularly important [23, 24]. A recent review of 26 qualitative and quantitative studies [23] reported BE factors at three levels being associated with PA in older adults, including campus (e.g., aesthetics, recreation amenities), building (e.g., ground-level housing, private dwellings), and fixtures (e.g., indoor hallways, ramps, accessible stairwells). Thus, more high-quality research is needed on the potential role of indoor environments in the promotion and facilitation of PA in older adults.

Although a few associations with BE attributes were identified for cycling, they were insufficiently studied compared to other PA outcomes. This lack of research may be due to the higher physical capacity required by older adults to participate in cycling, lack of cycling infrastructure, as well as a higher perceived risk of injury [51, 62]. In general, cycling allows for greater distance travelled, thereby increasing one’s neighbourhood radius, and offers greater benefits for older adults’ mobility, independence, and participation in social activities and their ability to maintain stronger social ties [51, 63]. Therefore, further research into BE attributes important for cycling in older adults is needed. For instance, the promotion of tricycles, proximity of multi-use paths, and having sufficient storage for tricycles in communal housing (including building rental programs) could be further explored.

There were several differences in the findings between ours and recent umbrella reviews on this topic. Across 16 systematic reviews (with search strategies including ≥2 bibliographic databases and including peer-reviewed and non-peer reviewed literature), Prince et al. [28] found null or mixed associations with PA outcomes for street connectivity, access to/availability of amenities, land-use mix, and public transport, all of which had positive associations with ≥1 PA outcome in our review. Some variation is likely due to the reviews in our umbrella review all being meta-analyses with definitive conclusions, whereas Prince et al. [28] included a mix of meta-analyses and narrative reviews. Across 11 peer-reviewed systematic, scoping, literature, and narrative reviews, Bonaccorsi et al. [29] reported positive associations with total PA (in ≥1 review) for land-use mix, street connectivity, and street lighting, whereas our review did not find these associations. Several methodological differences between reviews should be considered when comparing findings. Prince et al. [28] and Bonaccorsi et al. [29] included a wider range of review types and individual reviews published >10 years ago. Bonaccorsi et al. [29] also included reviews with participants ≥55 years-old, and Prince et al. [28] extracted data from primary studies of older adults (≥65 years-old) from reviews of all age-groups. Findings from Prince et al. [28] and Bonaccorsi et al. [29] can thus be seen as reflecting the broader scope of evidence available on the topic, with findings from our umbrella review focusing on the best and most up-to-date evidence for older adults ≥60 years-old.

### Strengths, Limitations, and Future Research

Following the JBI umbrella review methodology is a major strength of this umbrella review as it enhanced rigor and reduced potential bias. The screening process (i.e., titles and abstracts, full-text articles), critical appraisal assessments, and data extraction were independently performed by two reviewers. Having an experienced librarian create the search strategy is also known to improve quality of reviews and meta-analysis [64]. Including three meta-analyses in this review is a strength, as it allowed for a conservative quantitative synthesis of evidence and definitive conclusions (i.e., significant/non-significant), despite the inability to pool effect sizes [42]. Inclusion of only best-evidence systematic reviews allows greater confidence in findings.

As our umbrella review encompasses research published up to 2017, good quality systematic reviews are needed to summarize the most recent evidence. The primary studies were almost exclusively cross-sectional; therefore, we cannot make claims around causality. An important direction for future research is to conduct more longitudinal and experimental studies to allow for causal inferences. Fortunately, at least one quasi-experimental study is underway [65]. The most effective interventions may be those designed based on consistent correlates in the literature [52], and the best- and most-up-to-date-evidence. Further, multiple co-occurring interventions (including relevant programming) may be required to change PA [52].

Including only reviews published in English and excluding “grey” literature constitutes a limitation as reviews may have been missed. That being said, we did not specifically include grey literature as it is not peer-reviewed and therefore was not appropriate to include as best-evidence [39, 40]. Further, a large proportion of primary studies were conducted in North America and European countries, and thus more research is needed in other continents.

The findings compiled in this umbrella review reflect a limited range of BE attributes which could influence PA in older adults. Potential factors to investigate in future research include the presence of community, building, and/or personal gardens for growing both vegetables and flowers, which may promote healthy eating and physical movement. As noted previously, more research into how buildings (e.g., retirement communities, malls) can be better designed to support PA in older adults (e.g., attractive, brightly-lit and safe stairwells; long hallways for indoor walking), and how programming can be introduced to support use of these spaces (e.g., stair-prompt and health-promoting signage) is needed. Further, roles of various climates (e.g., ice and snow) and solutions promoting PA among older adult populations in these contexts should be considered, as many older adults do not venture outside in the winter due to fear of falling [21, 66].

Finally, the included reviews did not seek to review in-depth the optimal thresholds at which BE attributes have meaningful impacts on older adults’ PA. As threshold details are useful for including in guidelines [60, 67–69] to provide specific real-world guidance to professionals responsible for (re)designing communities, future systematic reviews should consider including an in-depth summary of these details.

### Conclusion

In this umbrella review, we provided a concise summary of the best and most up-to-date evidence on BE attributes that are important for facilitating PA in older adults (≥60 years). Several BE categories are associated with at least one type of PA. These include walkability, residential density and urbanization, street connectivity, access to/availability of services/destinations, pedestrian/cycling infrastructure and streetscape, aesthetics and cleanliness/order, and safety and traffic. Those involved in (re)designing age-friendly communities, such as policymakers, urban planners, architects, and developers, can use this best-evidence information.
