# Supplementary material for: An Umbrella Review of the Best and Most Up-to-Date Evidence on the Built Environment and Physical Activity in Older Adults ≥60 Years
Source: Public Health Rev. 2023 Mar 10;44:1605474. doi: 10.3389/phrs.2023.1605474 (PMC10037345; doi:10.3389/phrs.2023.1605474)
Supplement: Supplementary file 1 [file Table1.pdf]

Supplementary Material S1. Search strategies by database and results of initial search.

Database: Medline via OVID (1946 - July 30, 2020)
Total results: 152

Line number	Search terms	Number of results
1	environment design/ or built environment/	6807
2	social planning/ or city planning/	4953
3	urbanization/	6798
4	transportation/ or motor vehicles/ or automobiles/ or motorcycles/ or railroads/	26434
5	cities/ or residence characteristics/ or exp housing/	83616
6	public facilities/ or exp "sports and recreation facilities"/	1216
7	architecture/ or "facility design and construction"/ or architectural accessibility/	12341
8	("built environment*" or "synthetic environment*" or "physical environment*" or "community environment*" or "man-made environment*" or "neighbo?rhood environment*" or "created environment*" or "artificial landscape*" or "urban environment*" or "city environment*" or "urban design*" or "urban form" or "built form" or "community design*" or urbaniz* or "city plan*" or "environment* design*" or "landscape design*" or "landscape architecture" or "building design*" or "site design*" or "public space*" or "public amenit*" or streetscape* or "street design*" or "urban plan*" or suburb* or transportation or commut* or transit or walkability or "recreational facilit*" or "green space*" or park or parks or garden* or "bike lane*" or "cycl* lane*" or sidewalk* or "multi-use trail*" or "multiuse trail*" or "walking trail*" or "hiking trail*" or "biking trail*" or "cycling trail*" or "multi-use path*" or "multiuse path*" or subway* or train or trains or light-rail or bus or buses or busing).mp.	209670
9	or/1-8	307948
10	exp exercise/ or exp physical conditioning, human/ or exp running/ or exp walking/	214898
11	tai ji/ or yoga/	3834

12	exp sports/ or exp athletic performance/ or exp physical fitness/ or exp martial arts/ or exp racquet sports/ or exp running/ or exp snow sports/ or exp water sports/	183973
13	exp recreation/ or exp horticulture/ or exp "play and playthings"/	208329
14	"physical* activ*".mp.	118556
15	(exercise* or sport* or walk* or run* or jog* or swim* or dive* or diving or bike* or biking or cycl* or "spin* class*" or "physical* fit*" or "resistance train*" or "endurance train*" or "interval train*" or HIIT or yoga or "tai chi" or "tai ji" or pilates or dance* or dancing or "weight lift*" or badminton or tennis or squash or bowling or football or rugby or netball or basketball or volleyball or "volley ball" or soccer or hockey or ski or skis or skier* or skiing or snowboard* or boxing or golf* or ((horse* or equine) adj2 (ride* or riding or jumping))).mp.	2819088
16	("recreation* activit*" or "leisure activit*" or camp* or hobby or hobbies or garden* or horticulture or backpacking or hiking or camping or canoeing or "board game*" or chess or "playing cards" or surf* or billiards or snooker or sailing or boating or shuffleboard or fishing or "roller skat*" or rollerskat* or "roller blad*" or rollerblad*).mp.	1575895
17	sedentary behavior/	9387
18	((sedentary adj2 (behavio?r* or lifestyle*)) or (inactiv* or "low activity")).mp.	343523
19	or/10-18	4491224
20	aged/ or "aged, 80 and over"/	3119123
21	(elders or elderly or geriatric* or "old age*" or (seniors not "high school") or "older adult*" or centenarian* or nonagenarian* or octogenarian* or septuagenarian* or sexagenarian* or dottering or decrepit or tottering or overaged or "oldest old").mp.	419971
22	Geriatrics/	29985
23	gerontolog*.mp.	7467
24	or/20-23	3250375
25	9 and 19 and 24	8842
26	Technology Assessment, Biomedical/ or meta-analysis/ or "systematic review"/	204272

27	(((systematic or scoping or synthesis or umbrella or integrative or rapid or comprehensive or meta or realist or concept* or evidence or narrative or literature) adj3 (review* or overview* or analys?s or synthes?s or ethnography or study or studies or map or maps or mapped or mapping)) or meta-analys?s).ti,kf,pt.	698515
28	("health technolog* assessment*" or HTA or HTAs).ti,kf,pt.	2380
29	26 or 27 or 28	728746
30	25 and 29	152

Database: EMBASE via OVID (1974 - July 30, 2020)
Total results: 325

Line number	Search terms	Number of results
1	exp environmental planning/	13634
2	exp urban area/ or city/ or city planning/ or urbanization/	119889
3	exp "land use"/	139503
4	social aspect/ or demography/	306942
5	exp "traffic and transport"/ or exp motor vehicle/ or exp traffic/	213089
6	"construction work and architectural phenomena"/ or architectural barrier/ or architecture/ or housing/	48128
7	recreational park/	891
8	("built environment*" or "synthetic environment*" or "physical environment*" or "community environment*" or "man-made environment*" or "neighbo?rhood environment*" or "created environment*" or "artificial landscape*" or "urban environment*" or "city environment*" or "urban design*" or "urban form" or "built form" or "community design*" or urbaniz* or "city plan*" or "environment* design*" or "landscape design*" or "landscape architecture" or "building design*" or "site design*" or "public space*" or "public amenit*" or streetscape* or "street design*" or "urban plan*" or suburb* or transportation or commut* or transit or walkability or "recreational facilit*" or "green space*" or park or parks or garden* or "bike lane*" or "cycl* lane*" or sidewalk* or "multi-use trail*" or "multiuse trail*" or "walking trail*" or "hiking trail*" or "biking trail*" or "cycling trail*" or "multi-use path*" or	245519

	"multiuse path*" or subway* or train or trains or light-rail or bus or buses or busing).mp.	
9	or/1-8	915644
10	exp exercise/	340741
11	exp muscle exercise/	13397
12	fitness/	37080
13	exp physical activity/ or exp walking/	420565
14	exp sport/ or exp atheltics/ or exp ombat sport/ or exp racquet sport/	164567
15	exp kinesiotherapy/	78254
16	"physical activity, capacity and performance"/ or physical performance/ or training/	105358
17	"physical* activ*".mp.	217993
18	(exercise* or sport* or walk* or run* or jog* or swim* or dive* or diving or bike* or biking or cycl* or "spin* class*" or "physical* fit*" or "resistance train*" or "endurance train*" or "interval train*" or HIIT or yoga or "tai chi" or "tai ji" or pilates or dance* or dancing or "weight lift*" or badminton or tennis or squash or bowling or football or rugby or netball or basketball or volleyball or "volley ball" or soccer or hockey or ski or skis or skier* or skiing or snowboard* or boxing or golf* or ((horse* or equine) adj2 (ride* or riding or jumping))).mp.	3861085
19	exp recreation/ or exp recreational game/	75654
20	("recreation* activit*" or "leisure activit*" or camp* or hobby or hobbies or garden* or horticulture or backpacking or hiking or camping or canoeing or "board game*" or chess or "playing cards" or surf* or billiards or snooker or sailing or boating or shuffleboard or fishing or "roller skat*" or rollerskat* or "roller blad*" or rollerblad*).mp.	1747077
21	sedentary lifestyle/	14185

22	((sedentary adj2 (behavio?r* or lifestyle*)) or (inactiv* or "low activity")).mp.	440685
23	or/10-22	5967475
24	aged/ or very elderly/	2986384
25	(elders or elderly or geriatric* or "old age*" or (seniors not "high school") or "older adult*" or centenarian* or nonagenarian* or octogenarian* or septuagenarian* or sexagenarian* or dottering or decrepit or tottering or overaged or "oldest old").mp.	726672
26	geriatrics/	29815
27	gerontology/	3070
28	or/24-27	3155979
29	9 and 23 and 28	23943
30	meta analysis/	192268
31	"systematic review"/	255028
32	biomedical technology assessment/	14497
33	(((systematic or scoping or synthesis or umbrella or integrative or rapid or comprehensive or meta or realist or concept or evidence or narrative or literature) adj3 (review* or overview* or analys?s or synthes?s or ethnography or study or studies or map or maps or mapped or mapping)) or meta-analys?s).ti,kw,pt.	825642
34	("health technolog* assessment*" or HTA or HTAs).ti,kw,pt.	4034
35	or/30-34	977555993724
36	29 and 35	325

Database: Cumulative Index to Nursing and Allied Health Literature (CINAHL) Plus with Full Text via EBSCOhost (1936 - July 30, 2020)
Total results: 281

Line number	Search terms	Number of results
S1	(MH "Built Environment")	113
S2	(MH "Public Accommodation") OR (MH "Universal Design+") OR (MH "Architectural Accessibility+") OR (MH "Environmental Health")	11,245

S3	(MH "Residence Characteristics") OR (MH "Communities+") OR (MH "Geographic Factors+") OR (MH "Housing+") OR (MH "Public Spaces+")	87,461
S4	(MH "Transportation+") OR (MH "Motor Vehicles+")	24,429
S5	(MH "Architecture") OR (MH "Facility Design and Construction")	5,829
S6	("built environment*" or "synthetic environment*" or "physical environment*" or "community environment*" or "man-made environment*" or "neighbo#rhood environment*" or "created environment*" or "artificial landscape*" or "urban environment*" or "city environment*" or "urban design*" or "urban form" or "built form" or "community design*" or urbaniz* or "city plan*" or "environment* design*" or "landscape design*" or "landscape architecture" or "building design*" or "site design*" or "public space*" or "public amenit*" or streetscape* or "street design*" or "urban plan*" or suburb* or transportation or commut* or transit or walkability or "recreational facilit*" or "green space*" or park or parks or garden* or "bike lane*" or "cycl* lane*" or sidewalk* or "multi-use trail*" or "multiuse trail*" or "walking trail*" or "hiking trail*" or "biking trail*" or "cycling trail*" or "multi-use path*" or "multiuse path*" or subway* or train or trains or light-rail or bus or buses or busing)	55,093
S7	S1 OR S2 OR S3 OR S4 OR S5 OR S6	164,360
S8	(MH "Physical Activity") OR (MH "Exercise+") OR (MH "Aerobic Exercises+") OR (MH "Running+") OR (MH "Walking+") OR (MH "Muscle Strengthening+") OR (MH "Upper Extremity Exercises+") OR (MH "Leisure Activities") OR (MH "Recreation+") OR (MH "Dancing+") OR (MH "Physical Fitness+") OR (MH "Sports+") OR (MH "Animal Sports+") OR (MH "Aquatic Sports+") OR (MH "Athletic Training+") OR (MH "Contact Sports+") OR (MH "Racquet Sports+") OR (MH "Skating+") OR (MH "Skiing+") OR (MH "Snow Skiing+") OR (MH "Sports, Disabled+") OR (MH "Target Sports+") OR (MH "Team Sports+") OR (MH "Winter Sports+")	253,815
S9	(MH "Tai Chi") OR (MH "Yoga+")	11,220
S10	(MH "Pilates")	1,147
S11	(MH "Recreation+") OR (MH "Dancing+") OR (MH "Hobbies+") OR (MH "Play and Playthings+") OR (MH "Games+") OR (MH "Video Games+")	30,211

S12	(MH "Horticulture")	3,154
S13	"physical* activ*"	78,701
S14	(exercise* or sport* or walk* or run* or jog* or swim* or dive* or diving or bike* or biking or cycl* or "spin* class*" or "physical* fit*" or "resistance train*" or "endurance train*" or "interval train*" or HIIT or yoga or "tai chi" or "tai ji" or pilates or dance* or dancing or "weight lift*" or badminton or tennis or squash or bowling or football or rugby or netball or basketball or volleyball or "volley ball" or soccer or hockey or ski or skis or skier* or skiing or snowboard* or boxing or golf* or ((horse* or equine) N2 (ride* or riding or jumping)))	522,440
S15	("recreation* activit*" or "leisure activit*" or camp* or hobby or hobbies or garden* or horticulture or backpacking or hiking or camping or canoeing or "board game*" or chess or "playing cards" or surf* or billiards or snooker or sailing or boating or shuffleboard or fishing or "roller skat*" or rollerskat* or "roller blad*" or rollerblad*)	198,645
S16	(MH "Life Style, Sedentary+")	8,299
S17	((sedentary N2 (behavio#r* or lifestyle*)) or (inactiv* or "low activity"))	23,823
S18	S8 OR S9 OR S10 OR S11 OR S12 OR S13 OR S14 OR S15 OR S16 OR S17	770,593
S19	(MH "Aged") OR (MH "Aged, 80 and Over+")	828,520
S20	TI ((elders or elderly or geriatric* or "old age*" or (seniors not "high school") or "older adult*" or centenarian* or nonagenarian* or octogenarian* or septuagenarian* or sexagenarian* or dottering or decrepit or tottering or overaged or "oldest old")) OR AB ((elders or elderly or geriatric* or "old age*" or (seniors not "high school") or "older adult*" or centenarian* or nonagenarian* or octogenarian* or septuagenarian* or sexagenarian* or dottering or decrepit or tottering or overaged or "oldest old"))	187,741
S21	(MH "Geriatrics")	6,207
S22	S19 OR S20 OR S21	891,352
S23	S7 AND S18 AND S22	5,771
S24	(MH "Meta Analysis") or (MH "Literature Review+")	119,246

S25	TI ((((systematic or scoping or synthesis or umbrella or integrative or rapid or comprehensive or meta or realist or concept or evidence or narrative or literature) N3 (review* or overview* or analys?s or synthes?s or ethnography or study or studies or map or maps or mapped or mapping)) or meta-analys?s)) OR AB ((((systematic or scoping or synthesis or umbrella or integrative or rapid or comprehensive or meta or realist or concept or evidence or narrative or literature) N3 (review* or overview* or analys?s or synthes?s or ethnography or study or studies or map or maps or mapped or mapping)) or meta-analys?s))	298,831
S26	TI (("health technolog* assessment*" or HTA or HTAs)) OR AB (("health technolog* assessment*" or HTA or HTAs))	2,975
S27	S24 OR S25 OR S26	328,415
S28	S23 AND S27	290
S29	S23 AND S27 Limiters: Scholarly (Peer Reviewed) Journals	281

Database: Scopus via Elsevier (1976 - July 30, 2020)
Total results: 392

((TITLE-ABS-KEY ("built environment*" OR "synthetic environment*" OR "physical environment*" OR "community environment*" OR "man-made environment*" OR "neighborhood environment*" OR "neighbourhood environment*" OR "created environment*" OR "artificial landscape*" OR "urban environment*" OR "city environment*" OR "urban design*" OR "urban form" OR "built form" OR "community design*" OR urbaniz* OR "city plan*" OR "environment* design*" OR "landscape design*" OR "landscape architecture" OR "building design*" OR "site design*" OR "public space*" OR "public amenit*" OR streetscape* OR "street design*" OR "urban plan*" OR suburb* OR transportation OR commut* OR transit OR walkability OR "recreational facilit*" OR "green space*" OR park OR parks OR garden* OR "bike lane*" OR "cycl* lane*" OR sidewalk* OR "multi-use trail*" OR "multiuse trail*" OR "walking trail*" OR "hiking trail*" OR "biking trail*" OR "cycling trail*" OR "multi-use path*" OR "multiuse path*" OR subway* OR train OR trains OR light-rail OR bus OR buses OR busing)) AND ((TITLE-ABS-KEY ("physical* activ*")) OR (TITLE-ABS-KEY (exercise* OR sport* OR walk* OR run* OR jog* OR swim* OR dive* OR diving OR bike* OR biking OR cycl* OR "spin* class*" OR "physical* fit*" OR "resistance train*" OR "endurance train*" OR "interval train*" OR hiit OR yoga OR "tai chi" OR "tai ji" OR pilates OR dance* OR dancing OR "weight lift*" OR badminton OR tennis OR squash OR bowling OR football OR rugby OR netball OR basketball OR volleyball OR "volley ball" OR soccer OR hockey OR ski OR skis OR skier* OR skiing OR snowboard* OR boxing OR golf* OR ((horse* OR equine) W/2 (ride*

OR riding OR jumping))))) OR (TITLE-ABS-KEY ("recreation* activit*" OR "leisure activit*" OR camp* OR hobby OR hobbies OR garden* OR horticulture OR backpacking OR hiking OR camping OR canoeing OR "board game*" OR chess OR "playing cards" OR surf* OR billiards OR snooker OR sailing OR boating OR shuffleboard OR fishing OR "roller skat*" OR rollerskat* OR "roller blad*" OR rollerblad*)) OR (TITLE-ABS-KEY ((sedentary PRE/2 (behavior* OR behaviour* OR lifestyle*)) OR inactiv* OR "low activity"))) AND (TITLE-ABS-KEY (elders OR elderly OR geriatric* OR gerontolog* OR "old age*" OR (seniors AND NOT "high school") OR "older adult*" OR centenarian* OR nonagenarian* OR octogenarian* OR septuagenarian* OR sexagenarian* OR dottering OR decrepit OR tottering OR overaged OR "oldest old"))) AND (TITLE-ABS-KEY (((systematic OR scoping OR synthesis OR umbrella OR integrative OR rapid OR comprehensive OR meta OR realist OR concept OR evidence OR narrative OR literature) W/3 (review* OR overview* OR analysis OR analyses OR synthesis OR syntheses OR ethnography OR study OR studies OR map OR maps OR mapped OR mapping)) OR meta-analysis OR meta-analyses OR "health technology assessment*" OR hta OR htas))

Database: Environment Complete via EBSCOhost (1897 - July 30, 2020)
Total results: 30

Line number	Search Terms	Number of results
S1	("built environment*" or "synthetic environment*" or "physical environment*" or "community environment*" or "man-made environment*" or "neighbo#rhood environment*" or "created environment*" or "artificial landscape*" or "urban environment*" or "city environment*" or "urban design*" or "urban form" or "built form" or "community design*" or urbaniz* or "city plan*" or "environment* design*" or "landscape design*" or "landscape architecture" or "building design*" or "site design*" or "public space*" or "public amenit*" or streetscape* or "street design*" or "urban plan*" or suburb* or transportation or commut* or transit or walkability or "recreational facilit*" or "green space*" or park or parks or garden* or "bike lane*" or "cycl* lane*" or sidewalk* or "multi-use trail*" or "multiuse trail*" or "walking trail*" or "hiking trail*" or "biking trail*" or "cycling trail*" or "multi-use path*" or "multiuse path*" or subway* or train or trains or light-rail or bus or buses or busing)	442,007
S2	"physical* activ*" OR ((exercise* or sport* or walk* or run* or jog* or swim* or dive* or diving or bike* or biking or cycl* or "spin* class*" or "physical* fit*" or "resistance train*" or "endurance train*"	1,251,247

	or "interval train*" or HIIT or yoga or "tai chi" or "tai ji" or pilates or dance* or dancing or "weight lift*" or badminton or tennis or squash or bowling or football or rugby or netball or basketball or volleyball or "volley ball" or soccer or hockey or ski or skis or skier* or skiing or snowboard* or boxing or golf* or ((horse* or equine) N2 (ride* or riding or jumping)))) OR (("recreation* activit*" or "leisure activit*" or camp* or hobby or hobbies or garden* or horticulture or backpacking or hiking or camping or canoeing or "board game*" or chess or "playing cards" or surf* or billiards or snooker or sailing or boating or shuffleboard or fishing or "roller skat*" or rollerskat* or "roller blad*" or rollerblad*)) OR (((sedentary N2 (behavio#r* or lifestyle*)) or (inactiv* or "low activity")))	
S3	TI ((elders or elderly or geriatric* or "old age*" or (seniors not "high school") or "older adult*" or centenarian* or nonagenarian* or octogenarian* or septuagenarian* or sexagenarian* or dottering or decrepit or tottering or overaged or "oldest old")) OR AB ((elders or elderly or geriatric* or "old age*" or (seniors not "high school") or "older adult*" or centenarian* or nonagenarian* or octogenarian* or septuagenarian* or sexagenarian* or dottering or decrepit or tottering or overaged or "oldest old"))	17,007
S4	(TI ((((systematic or scoping or synthesis or umbrella or integrative or rapid or comprehensive or meta or realist or concept or evidence or narrative or literature) N3 (review* or overview* or analys?s or synthes?s or ethnography or study or studies or map or maps or mapped or mapping)) or meta-analys?s)) OR AB ((((systematic or scoping or synthesis or umbrella or integrative or rapid or comprehensive or meta or realist or concept or evidence or narrative or literature) N3 (review* or overview* or analys?s or synthes?s or ethnography or study or studies or map or maps or mapped or mapping)) or meta-analys?s))) OR (TI (("health technolog* assessment*" or HTA or HTAs)) OR AB (("health technolog* assessment*" or HTA or HTAs)))	80,863
S5	S1 AND S2 AND S3 AND S4	30

Database: Cochrane Library (1992 - July 30, 2020)
Total results: **114

**Only Cochrane Reviews and Protocols exported

Line Number	Search Terms
#1	[mh ^"environment design"] or [mh ^"built environment"]
#2	[mh ^"social planning"] or [mh ^"city planning"]
#3	[mh ^urbanization]
#4	[mh ^transportation] or [mh ^"motor vehicles"] or [mh ^automobiles] or [mh ^motorcycles] or [mh ^railroads]
#5	[mh ^cities] or [mh ^"residence characteristics"] or [mh housing]
#6	[mh ^"public facilities"] or [mh "sports and recreation facilities"]
#7	[mh ^architecture] or [mh ^"facility design and construction"] or [mh ^"architectural accessibility"]
#8	(built or synthetic or physical or community or man-made or neighbo?rhood or created or artificial or urban or city) NEAR/2 (landscape* or environment*):ti,ab,kw
#9	(urban or community or environment or landscape or building or site or street) NEAR/2 (design* or architectur*):ti,ab,kw
#10	urban form or "built form":ti,ab,kw
#11	urbaniz* or "city planning" or "city plan" or "urban plan" or "urban planning" or streetscape*:ti,ab,kw
#12	(public or green or park*) NEAR/2 (space* or amenit*):ti,ab,kw
#13	suburb*:ti,ab,kw
#14	transport* or transit or commut* or bikability or walkability or sidewalk* or "side-walk*" or train or trains or light-rail or subway or bus or buses or busing:ti,ab,kw
#15	park or parks or garden*:ti,ab,kw
#16	(bike* or biking or bicycle* or bicycling or walking or hiking or multi-use or multiuse) NEAR/2 (path* or path-way* or lane* or trail*):ti,ab,kw
#17	(recreation* or leisure or sport*) NEAR/2 facilit*:ti,ab,kw
#18	{OR #1-#17}
#19	[mh exercise] or [mh ^"physical conditioning, human"] or [mh ^running] or [mh ^walking]

#20	[mh ^"tai ji"] or [mh ^yoga]
#21	[mh sports] or [mh "athletic performance"] or [mh "physical fitness"] or [mh "martial arts"] or [mh "racquet sports"] or [mh "snow sports"] or [mh "water sports"] or [mh swimming]
#22	[mh recreation] or [mh horticulture] or [mh "play and playthings"]
#23	(exercise* or sport* or walk* or run* or jog* or swim* or dive* or diving or bike* or biking or cycl* or "spin class" or "spinning class" or HIIT or yoga or "tai chi" or "tai ji" or pilates or dance* or dancing or "weight lifting" or badminton or tennis or squash or bowling or football or rugby or netball or basketball or volleyball or "volley ball" or soccer or hockey or ski or skis or skier* or skiing or snowboard* or boxing or golf* or ((horse* or equine) NEAR/2 (ride* or riding or jumping))):ti,ab,kw
#24	(resistance or endurance or interval) NEAR/2 train*:ti,ab,kw
#25	physical* NEAR/1 (activ* or fit*):ti,ab,kw
#26	(recreation* or leisure or sport*) NEAR/1 activit*:ti,ab,kw
#27	(camp* or hobby or hobbies or garden* or horticulture or backpacking or hiking or camping or canoeing or "board game" or chess or "playing cards" or surf* or billiards or snooker or sailing or boating or shuffleboard or fishing or "roller skate" or "roller skating" or rollerskat* or "roller blade" or "roller blading" or rollerblad*):ti,ab,kw
#28	[mh ^"sedentary behavior"]
#29	(sedentary NEAR/2 (behavio?r* or lifestyle*)) or inactiv* or "low activity":ti,ab,kw
#30	{OR #19-#29}
#31	[mh ^aged] or [mh ^"aged, 80 and over"]
#32	((elders or elderly or geriatric* or "old age" or "old aged" or "old ages" or (seniors not "high school") or "older adult" or "older adults" or centenarian* or nonagenarian* or octogenarian* or septuagenarian* or sexagenarian* or dottering or decrepit or tottering or overaged or "oldest old")):ti,ab,kw
#33	[mh ^geriatrics]
#34	gerontolog*:ti,ab,kw
#35	{OR #31-#34}
#36	#18 AND #30 AND #35
