# Supplementary material for: An Umbrella Review of the Best and Most Up-to-Date Evidence on the Built Environment and Physical Activity in Older Adults ≥60 Years
Source: Public Health Rev. 2023 Mar 10;44:1605474. doi: 10.3389/phrs.2023.1605474 (PMC10037345; doi:10.3389/phrs.2023.1605474)
Supplement: Supplementary file 2 [file Table2.pdf]

Supplementary Material S2. Critical appraisal of the included systematic reviews.

	**Included Systematic Reviews (First author Year)**		
	**Barnett 2017**	**Cerin 2017**	**Van Cauwenberg 2018**
1. Is the review question clearly and explicitly stated?	Yes	Yes	Yes
2. Were the inclusion criteria appropriate for the review question?	Yes	Yes	Yes
3. Was the search strategy appropriate?	Yes	Yes	Yes
4. Were the sources and resources used to search for the studies adequate?	Yes	Yes	Yes
5. Were the criteria for appraising the studies appropriate?	Yes	Yes	Yes
6. Was critical appraisal conducted by two or more reviewers independently?	Yes	Yes	Yes
7. Were there methods to minimize errors in data extraction?	Yes	Yes	Yes
8. Were the methods used to combine studies appropriate?	Yes	Yes	Yes
9. Was the likelihood of publication bias assessed?	Yes	Yes	Yes
10. Were recommendations for policy and/or practice supported by the reported data?	Yes	Yes	Yes
11. Were the specific directives for new research appropriate?	Yes	Yes	Yes
12. Overall appraisal (include/exclude):	Include	Include	Include
13. Overall Score:	11	11	11

Note. Critical appraisals were completed using the Joanna Briggs Institute Critical Appraisal Tool.
